# Supplementary material for: Oxidative Stress Model of Lipopolysaccharide-Challenge in Piglets of Wuzhishan Miniature Pig
Source: Vet Sci. 2025 Jul 24;12(8):694. doi: 10.3390/vetsci12080694 (PMC12390522; doi:10.3390/vetsci12080694)
Supplement: Supplementary file 1 [file vetsci-12-00694-s001.zip › vetsci-3713714-supplementary.pdf]

Supplementary Tables

Supplementary Table S1. Body weight of piglets.

Body weight (kg)	Groups			
	Control	L-LPS	M-LPS	H-LPS
Day 0	4.25 ± 0.30	4.21 ± 0.50	4.14 ± 0.61	4.10 ± 0.42
Day 1	4.33 ± 0.80	3.98 ± 0.58	4.00 ± 0.16	3.70 ± 0.55
Day 2	4.55 ± 0.90	4.06 ± 0.84	3.80 ± 0.40	3.73 ± 0.49
Day 3	4.61 ± 1.04	4.06 ± 0.81	3.90 ± 0.48	3.76 ± 0.54
Day 4	4.81 ± 0.92	4.10 ± 1.02	3.99 ± 0.54	3.76 ± 0.85
Day 5	4.81 ± 1.10	4.29 ± 1.04	4.03 ± 0.75	3.82 ± 0.86
Day 6	5.11 ± 1.24	4.57 ± 1.25	4.28 ± 0.90	3.89 ± 1.40
Day 7	5.37 ± 1.32	4.80 ± 1.35	4.31 ± 0.78	3.96 ± 1.44

Supplementary Table S2. Effect of LPS on gut microbiota α-diversity on day 1.

α-diversity	Groups		Wilcoxon rank-sum test p-value
	Control	M-LPS	
Observed_species	11864.40 ± 605.00	12472.33 ± 1495.41	0.130
Shannon	8.12 ± 0.65	8.44 ± 0.26	0.540
Simpson	0.97 ± 0.02	0.98 ± 0.00	0.930
Chao1	12304.61 ± 613.44	12956.49 ± 1662.01	0.082

Supplementary Table S3. Effect of LPS on gut microbiota α-diversity on day 7.

α-diversity	Groups		Wilcoxon rank-sum test p-value
	Control	M-LPS	
Observed_species	5993.33 ± 866.76	11365.6 ± 745.41	0.0043
Shannon	5.54 ± 0.12	7.77 ± 0.41	0.0043
Simpson	0.89 ± 0.01	0.97 ± 0.01	0.0043
Chao1	6169.63 ± 854.79	11548.71 ± 781.45	0.0043

Supplementary Table S4. Effect of LPS on gut microbiota compositions on day 1.

Composition (%)	Groups		Mann Whitney U test *p*-value
	Control	M-LPS	
p__Spirochaetes	0.84 ± 0.42	1.64 ± 0.42	0.0062
p__Tenericutes	1.27 ± 0.88	0.24 ± 0.33	0.0176
p__Firmicutes	44.34 ± 10.23	42.29 ± 4.44	0.1441
p__Euryarchaeota	0.81 ± 0.27	0.58 ± 0.41	0.2733
p__Bacteroidetes	23.6 ± 14.13	22.45 ± 5.39	0.3613
p__Proteobacteria	2.60 ± 0.85	2.86 ± 1.20	0.7150
p__Actinobacteria	1.07 ± 0.44	1.24 ± 0.98	0.8551
g__Bacteroides	4.60 ± 0.51	6.94 ± 1.45	0.0062
g__Mycoplasma	1.20 ± 0.88	0.17 ± 0.30	0.0176
g__Oscillibacter	2.19 ± 0.89	1.41 ± 0.67	0.1003
g__Blautia	1.01 ± 0.45	1.14 ± 0.09	0.1003
g__Lachnoclostridium	0.77 ± 0.45	1.07 ± 0.18	0.1003
g__Parabacteroides	2.62 ± 1.51	1.41 ± 0.18	0.2012
g__Ruminococcus	2.25 ± 0.61	3.20 ± 0.87	0.2012
g__Clostridium	8.33 ± 4.17	5.13 ± 1.96	0.2733
g__Sphaerochaeta	0.51 ± 0.33	0.87 ± 0.44	0.2733
g__Dorea	0.54 ± 0.45	0.56 ± 0.16	0.3613
g__Subdoligranulum	0.63 ± 0.52	0.39 ± 0.06	0.4652
g__Roseburia	1.09 ± 0.82	1.06 ± 0.35	0.5839
g__Faecalibacterium	0.74 ± 0.18	0.79 ± 0.26	0.5839
g__Phascolarctobacterium	0.78 ± 0.20	0.90 ± 0.46	0.5839
g__Alistipes	0.94 ± 0.66	0.71 ± 0.28	0.5839
g__Coprococcus	0.52 ± 0.34	0.45 ± 0.17	0.5839
g__Negativibacillus	0.55 ± 0.50	0.27 ± 0.17	0.5839
g__Eubacterium	1.59 ± 0.39	1.61 ± 0.89	0.7150
g__Treponema	0.18 ± 0.09	0.54 ± 0.27	0.0285
g__Escherichia	0.14 ± 0.07	0.58 ± 0.45	0.1003
g__Flavonifractor	0.44 ± 0.10	0.63 ± 0.27	0.1441
s__Oscillibacter_sp._CAG:241	0.55 ± 0.46	0.07 ± 0.03	0.0446
s__Firmicutes_bacterium_CAG:176	0.54 ± 0.41	0.10 ± 0.06	0.0679
s__Parabacteroides_sp._CAG:409	1.74 ± 1.32	0.06 ± 0.03	0.1003
s__Phascolarctobacterium_succinatutens	0.75 ± 0.21	0.84 ± 0.45	0.5839
s__Negativibacillus_massiliensis	0.55 ± 0.50	0.27 ± 0.17	0.5839
s__Ruminococcus_sp._CAG:177	0.54 ± 0.39	0.74 ± 0.64	0.7150
s__Escherichia_coli	0.14 ± 0.07	0.54 ± 0.41	0.1441
s__Ruminococcus_bromii	0.23 ± 0.08	0.72 ± 0.65	0.4652

Supplementary Table S5. Effect of LPS on gut microbiota compositions on day 7.

Composition (%)	Groups		Mann Whitney U test *p*-value
	Control	M-LPS	
p__Bacteroidetes	66.60 ± 4.01	30.93 ± 14.06	0.0062
p__Firmicutes	18.29 ± 2.26	32.11 ± 13.62	0.0062
p__Proteobacteria	0.69 ± 0.27	1.25 ± 0.48	0.0106
p__Actinobacteria	0.86 ± 0.49	0.36 ± 0.20	0.0285
p__Spirochaetes	10.42 ± 0.81	3.09 ± 0.00	0.0062
p__Euryarchaeota	1.70 ± 0.49	0.03 ± 0.01	0.0062
g__Clostridium	0.99 ± 0.22	4.04 ± 0.81	0.0062
g__Collinsella	0.67 ± 0.50	0.13 ± 0.12	0.0285
g__Oscillibacter	0.58 ± 0.38	1.11 ± 0.17	0.0446
g__Prevotella	35.16 ± 25.74	16.63 ± 11.23	0.2012
g__Faecalibacterium	1.73 ± 0.79	1.35 ± 0.37	0.3613
g__Phascolarctobacterium	1.07 ± 0.69	0.71 ± 0.28	0.4652
g__Bacteroides	25.23 ± 24.62	5.65 ± 1.31	0.7150
g__Roseburia	1.30 ± 1.11	1.31 ± 0.32	1.0000
g__Ruminococcus	0.33 ± 0.14	1.70 ± 0.66	0.0062
g__Eubacterium	0.33 ± 0.22	0.95 ± 0.24	0.0062
g__Blautia	0.35 ± 0.03	1.11 ± 0.20	0.0062
g__Lachnoclostridium	0.18 ± 0.01	0.71 ± 0.35	0.0062
g__Treponema	0.01 ± 0.00	1.10 ± 0.35	0.0062
s__Bacteroides_thetaiotaomicron	1.23 ± 1.15	0.15 ± 0.04	0.0062
s__Lactobacillus_amylovorus	0.71 ± 1.02	0.00 ± 0.00	0.0062
s__Prevotella_copri	16.68 ± 11.7	3.34 ± 2.66	0.1003
s__Faecalibacterium_prausnitzii	1.04 ± 0.44	0.71 ± 0.22	0.2012
s__Bacteroides_fragilis	0.79 ± 0.75	0.20 ± 0.07	0.2733
s__Phascolarctobacterium_succinatutens	1.06 ± 0.68	0.68 ± 0.28	0.4652
s__Prevotella_sp._CAG:386	2.71 ± 2.54	1.67 ± 1.46	0.5839
s__Prevotella_sp._Marseille-P4119	0.52 ± 0.47	0.23 ± 0.16	0.5839
s__Firmicutes_bacterium_CAG:110	0.02 ± 0.01	0.73 ± 0.53	0.0062
s__Prevotella_sp._P5-92	0.23 ± 0.21	1.22 ± 0.64	0.0285
s__Prevotella_sp._P2-180	0.22 ± 0.20	0.97 ± 0.63	0.1003

Supplementary Figures

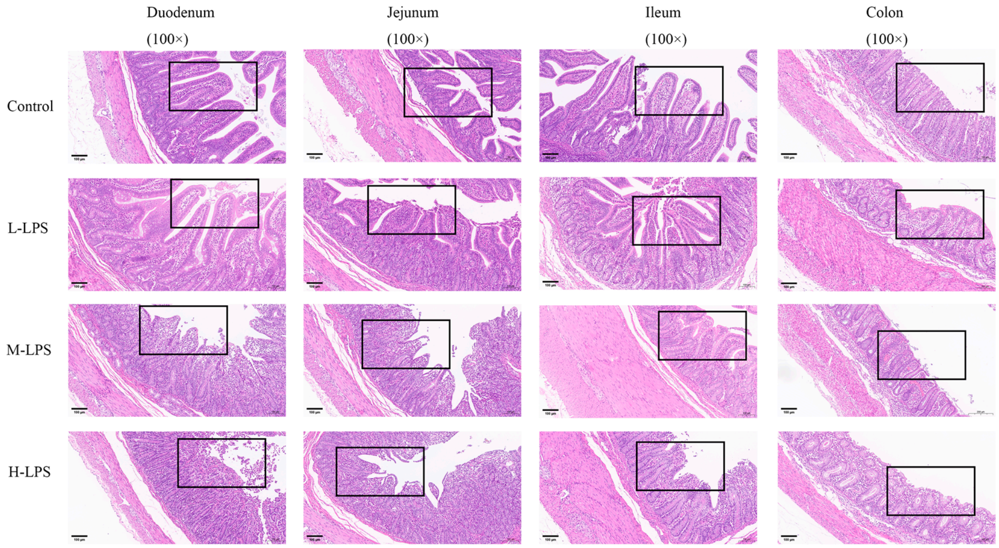

Supplementary Figure S1. Representative H&E staining of small intestine sections. Scale bar: 100 μm. Black boxes outlines typical areas.

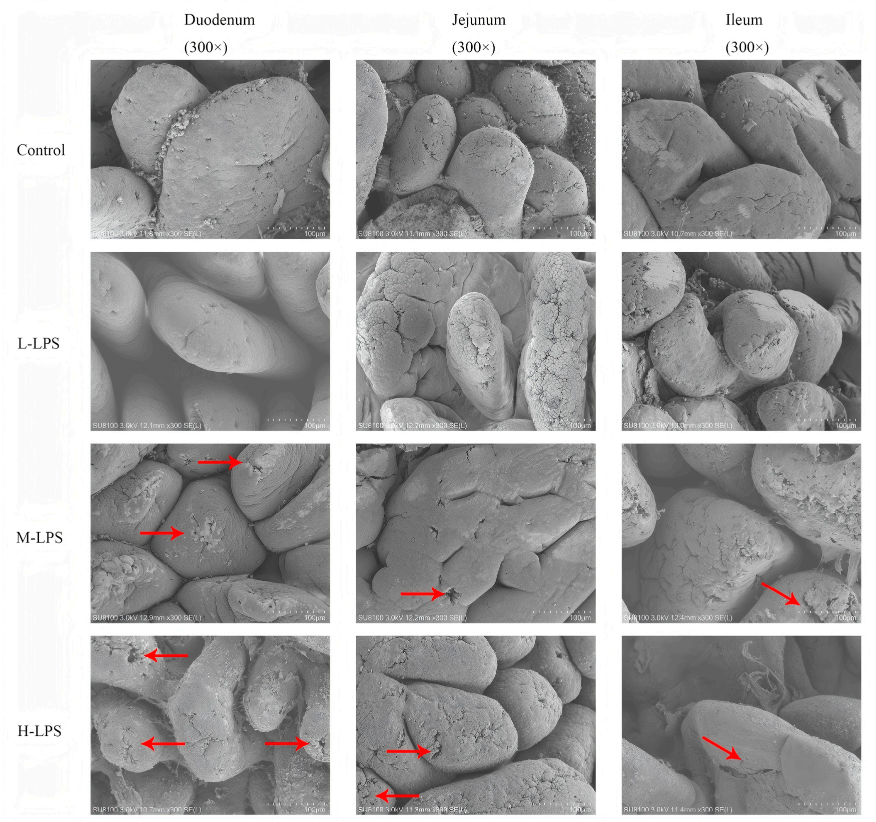

Supplementary Figure S2. Representative SEM images of small intestine sections. Scale bar: 100 μm. Red arrows indicates damaged intestinal villi.

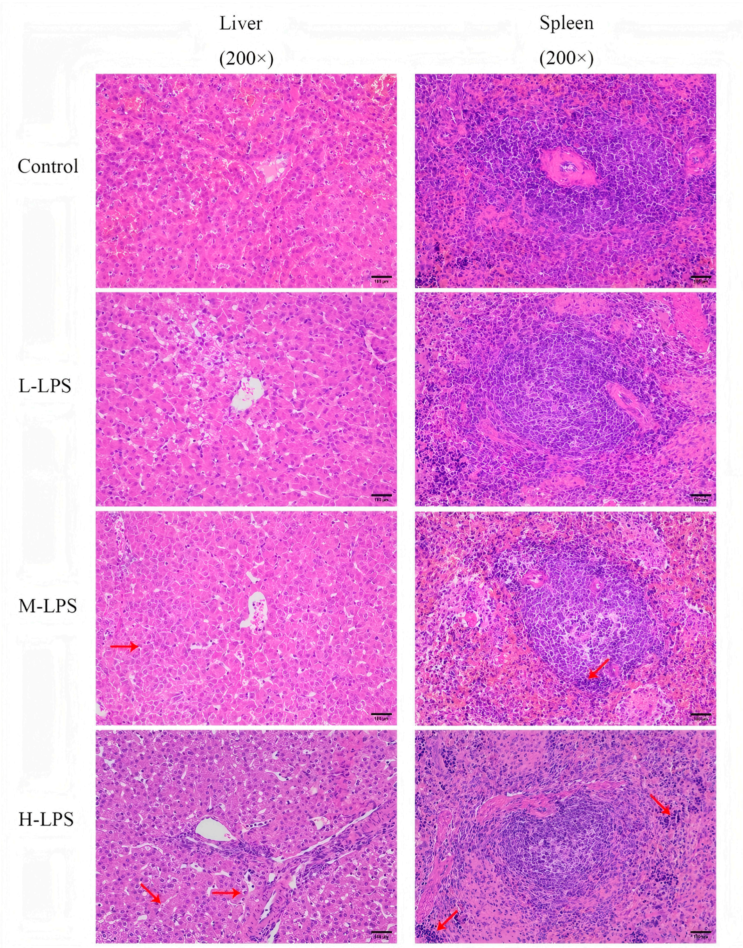

Supplementary Figure S3. Representative H&E staining of liver and spleen sections. Scale bar: 100 μm. Red arrows indicates lesions areas.

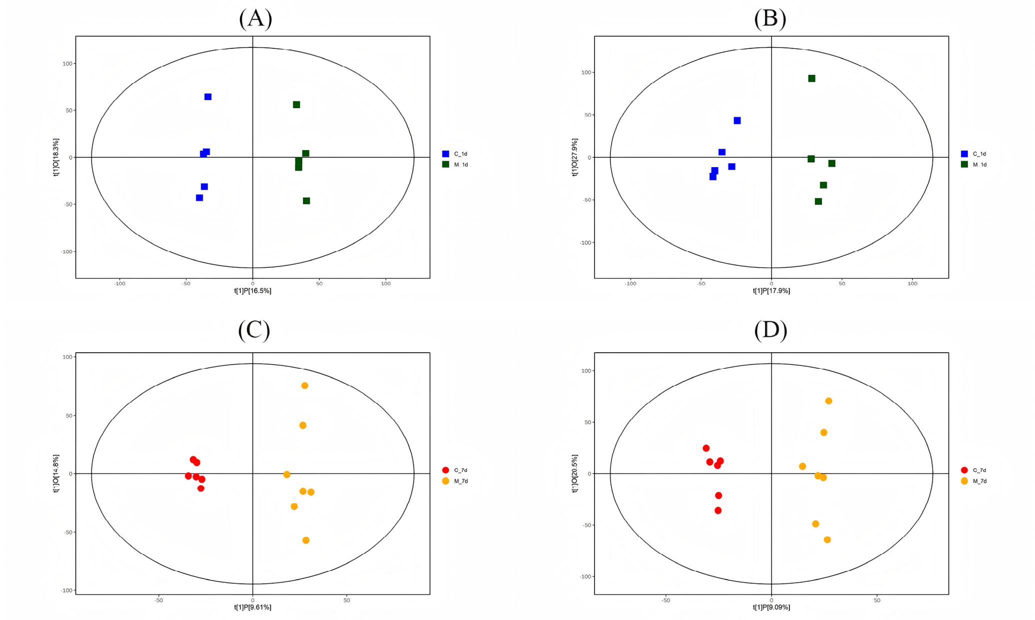

Supplementary Figure S4. Orthogonal partial least squares-discriminant analysis (OPLS-DA) score plots of the serum metabolites between the control and M-LPS groups. (**A**) In ESI$^-$ mode (1 d); (**B**) In ESI$^+$ mode (1 d); (**C**) In ESI$^-$ mode (7 d); (**D**) In ESI$^+$ mode (7 d).

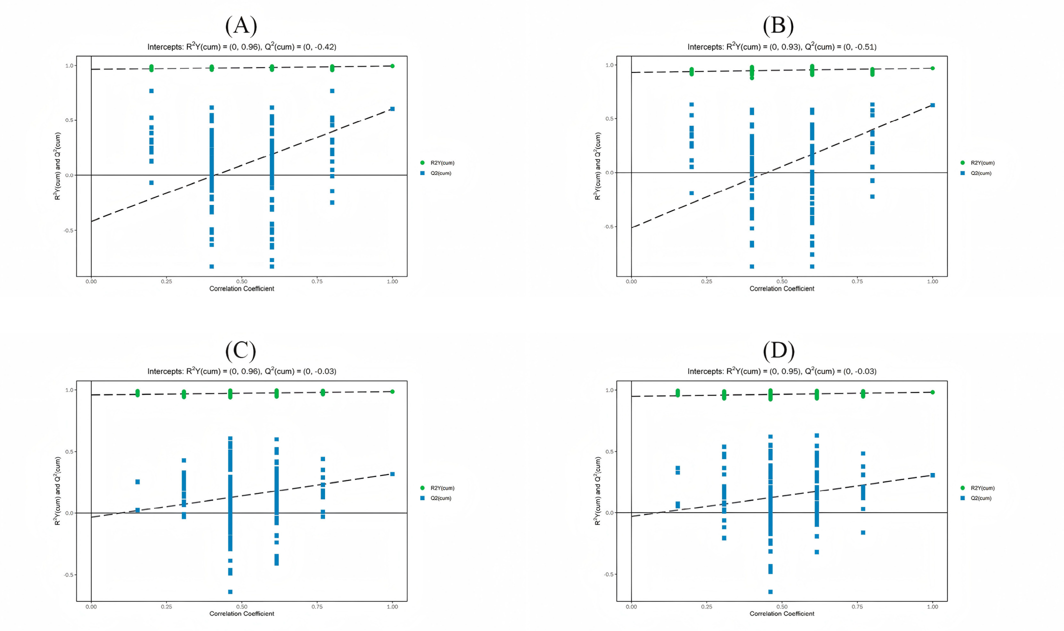

Supplementary Figure S5. OPLS-DA permutation test plots of the serum metabolites between the control and M-LPS groups. (**A**) In ESI$^-$ mode (1 d); (**B**) In ESI$^+$ mode (1 d); (**C**) In ESI$^-$ mode (7 d); (**D**) In ESI$^+$ mode (7 d).
